# Effects of Covid-19 restrictions on IPS service delivery in Northern Norway

**DOI:** 10.1192/j.eurpsy.2022.503

**Published:** 2022-09-01

**Authors:** S. Wittlund

**Affiliations:** Nordlands Hospital, Bodø, Regional Competence Centre For Work And Mental Health (rkaph), BODØ, Norway

**Keywords:** individual placement and support, IPS, Covid-19, Employment specialist

## Abstract

**Introduction:**

Individual Placement and Support (IPS) is an evidence-based supported employment program that helps people with severe mental illness to achieve steady meaningful employment in competitive mainstream jobs. Employment specialists are an integral part of IPS service delivery. The primary goal of an employment specialist is to help IPS users obtain competitive employment by providing targeted job development and ongoing support to workers and employers for as long as it is required.

**Objectives:**

This study aims to investigate the impact of the covid-19 restrictions on the delivery of IPS services in Northern Norway and how this may have affected the employment specialists’ perception their work environment.

**Methods:**

We conducted four phases of a longitudinal work environment panel survey with the IPS employment specialists in Northern Norway. Phase 1: January-February 2020 (pre-covid), phase 2: June-July 2020 (during covid) and phase 3: October-November 2020 (during covid) were not related to covid and collected data on fourteen work environment indicators. Phase 4: October 2020 was a covid specific survey and collected data about the impact of covid-19 restrictions on IPS service delivery.

**Results:**

Employment specialists perceived that they had less collaborative engagement with clinical teams and employers after covid-19 restrictions were introduced. This was accompanied by a significant decline in four of the employment specialists’ work environment indicators.

**Conclusions:**

The covid-19 restrictions appear to have created obstacles for IPS service delivery in Northern Norway. These challenges may have negatively impacted the employment specialists’ perception of their work environment, creating job dissatisfaction and potentially increasing employee attrition.

**Disclosure:**

No significant relationships.

